# Feasibility of using surgical implantation methods for acoustically tagging alewife (*Alosa pseudoharengus*) with V5 acoustic transmitters

**DOI:** 10.1371/journal.pone.0241118

**Published:** 2020-11-30

**Authors:** Elizabetha Tsitrin, Montana F. McLean, A. Jamie F. Gibson, David C. Hardie, Michael J. W. Stokesbury

**Affiliations:** 1 Department of Biology, Acadia University, Wolfville, Nova Scotia, Canada; 2 Department of Fisheries and Oceans Canada, Dartmouth, Nova Scotia, Canada; Pacific Northwest National Laboratory, UNITED STATES

## Abstract

Anadromous alewives (*Alosa pseudoharengus*) are abundant in the Canadian Maritimes, where they support lucrative commercial fisheries. Little is known about their coastal movement, and their potential to interact with anthropogenic structures. Acoustic telemetry can provide detailed information on the spatiotemporal distribution and survival of fishes in coastal areas, using information transmitted from tagged fishes and recorded by moored receivers. However, few acoustic telemetry studies have been performed on clupeids as they are extremely sensitive to handling, and are often compromised by surgical tag implantation. This research assesses the feasibility of a surgical tagging protocol using novel High Residency acoustic tags in alewives, and establishes a baseline of short-term tagging effects. Alewives from the Gaspereau River population were tagged between 2018 (n = 29) and 2019 (n = 96) with non-transmitting models of Vemco/Innovasea V5 HR tags. Tagging effects were evaluated based on recovery rate, reflex impairment, and necropsy-based health assessments. Alewives responded well to tagging, with low mortality (3%) and no observed instances of tag shedding 72 hours post-surgery. The use of sutures to close the incision site had no effect on recovery times. Water temperature and spawning condition had the greatest effect on the behavioural response of fish to tagging. Our findings suggest that, with proper handling and smaller acoustic tags, telemetry studies on alewives are feasible.

## Introduction

Determining how animals move and interact with their environment is fundamental to understanding their ecology. Information gathered about migration patterns, habitat use, physiology and energetics, population dynamics, survival, and behaviour are also essential components of management and conservation science [1]. Acoustic telemetry has become a popular tool for studying the spatial and temporal movements of individuals in aquatic ecosystems, where direct observation is challenging. However, the surgical implantation of acoustic tags can be stressful on the animal, resulting in physical injuries to internal organs [2], scale loss following the removal of scales for tagging [3], sublethal effects of stress on physiology and behaviour [4,5], increased susceptibility to disease [6], and reduced swimming performance [7].

Clupeidae, which includes herrings, shads, and alewives, is a family often considered sensitive to handling [8–10]. Clupeids are widely distributed along the North Atlantic seaboard of Canada and the United States, and support lucrative commercial and recreational fisheries throughout their range [11,12]. Anadromous species such as alewife (*Alosa pseudoharengus*) also serve an important ecological role as vectors for marine nutrient transport to inland waters [13–15]. Few tagging studies have been performed on clupeids, with most of these focusing on the Pacific herring (*Clupea pallasii*) [16,17]. Even fewer data exist for alewife [18,19]. This is in part because clupeids are susceptible to scale loss, infection, and behavioural changes when handled [9,10,20]. The limited scope, varying methods, and small sample sizes associated with past studies raise questions related to the feasibility of acoustic tagging of clupeids. However, recent developments in acoustic technology, such as the decrease in tag size, could improve methods of surgical implantation of acoustic transmitters in these fragile fishes.

Advancing the tagging techniques requires empirical assessment to ensure that data generated are biologically relevant (i.e., no long-term behavioural or physiological consequences), and that fish remain viable after tagging. Quantifying tag effects *in situ* is difficult because animals cannot usually be directly observed. Factors such as tag design, handling methods, ambient environmental conditions, and the maturity, physical condition, and size of the fish can affect tagging outcomes [21,22], and latent effects can result in the mortality of individuals previously deemed viable [6,16]. Consequently, there is a need for a method of assessing vitality in tagged fish that can help predict post-tagging survival. One such method, which provides both a quick and non-invasive procedure for quantifying fish stress before release, is the use of reflex impairment indices, or Reflex Action Mortality Predictors (RAMP) [23–25].

Reflexes are involuntary, stereotyped movements induced by a peripheral stimulus, and in fishes are linked to fitness outcomes such as reduced growth, impaired predator evasion, and delayed mortality [25]. Greater reflex impairment scores are associated with physiological exhaustion and reduced vitality in many species [23–27]. Therefore, RAMP may be an effective tool for rapid, real-time assessment of fish stress, with applications in catch-and-release fisheries, bycatch, and tracking studies [24,26–28].

In this study we examined the feasibility of surgically implanting acoustic tags in alewives from the Gaspereau River population in Nova Scotia, Canada, using V5 High Residency tags manufactured by Vemco/Innovasea (Bedford, Nova Scotia, Canada). The study was conducted over two field seasons. In 2018, trials were conducted to a) determine the optimal concentration of MS-222 for inducing surgical anesthesia in alewife, and b) test the short-term effects on recovery time and survival based on whether or not sutures were used to close the incision. In 2019, short-term physiological and environmental effects of tagging were assessed using reflex impairment and necropsy. The discussion provides recommendations for further study to better understand sublethal and latent effects of tagging and handling on clupeids.

## Methods

### Study area

**Fig 1:**
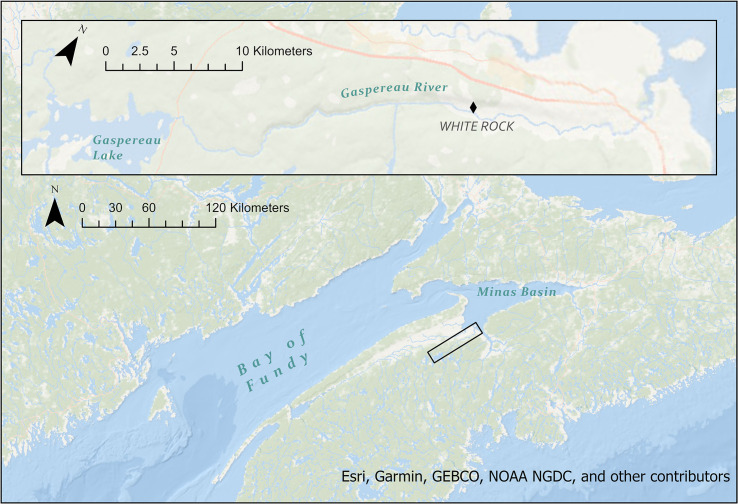
Map of study area, showing Minas Basin and an inset of Gaspereau River and Gaspereau Lake. The tagging site at White Rock Fish Ladder is designated by a black diamond.

Gaspereau River in Kings County, Nova Scotia, is part of an extensive watershed managed by the province for hydroelectricity generation, and supports two generating stations. Along with several large rivers in Nova Scotia, Gaspereau River drains into the Minas Basin–a macrotidal estuary at the head of the Bay of Fundy (Fig 1) [29,30]. This area is renowned for extreme tidal amplitudes ranging from 5 to 16 m [30]. The Bay of Fundy is an important area for biodiversity, supporting rich assemblages of benthic invertebrates, migratory shorebirds, fish, and marine mammals [31,32], and includes important spawning grounds for species like Atlantic herring.

Two field seasons were conducted in 2018 and 2019. Between 23 May and 13 June 2018, alewives were tagged with non-transmitting (dummy) acoustic tags, and were used for testing anesthesia concentrations, and to compare recovery time between incisions closed with sutures and those left open. Between 19 May and 25 June 2019, alewife were tagged with dummy acoustic transmitters to assess reflex impairment and internal physiology. Animal capture, holding and tagging procedures were analogous between the two tagging years, unless otherwise stated. This study was carried out in accordance with the recommendations of the Canadian Council on Animal Care for the Marking and Tagging of Finfish. The protocol was approved by the Acadia University Animal Care Committee (protocol 07–18).

### Fish capture and handling

**Fig 2:**
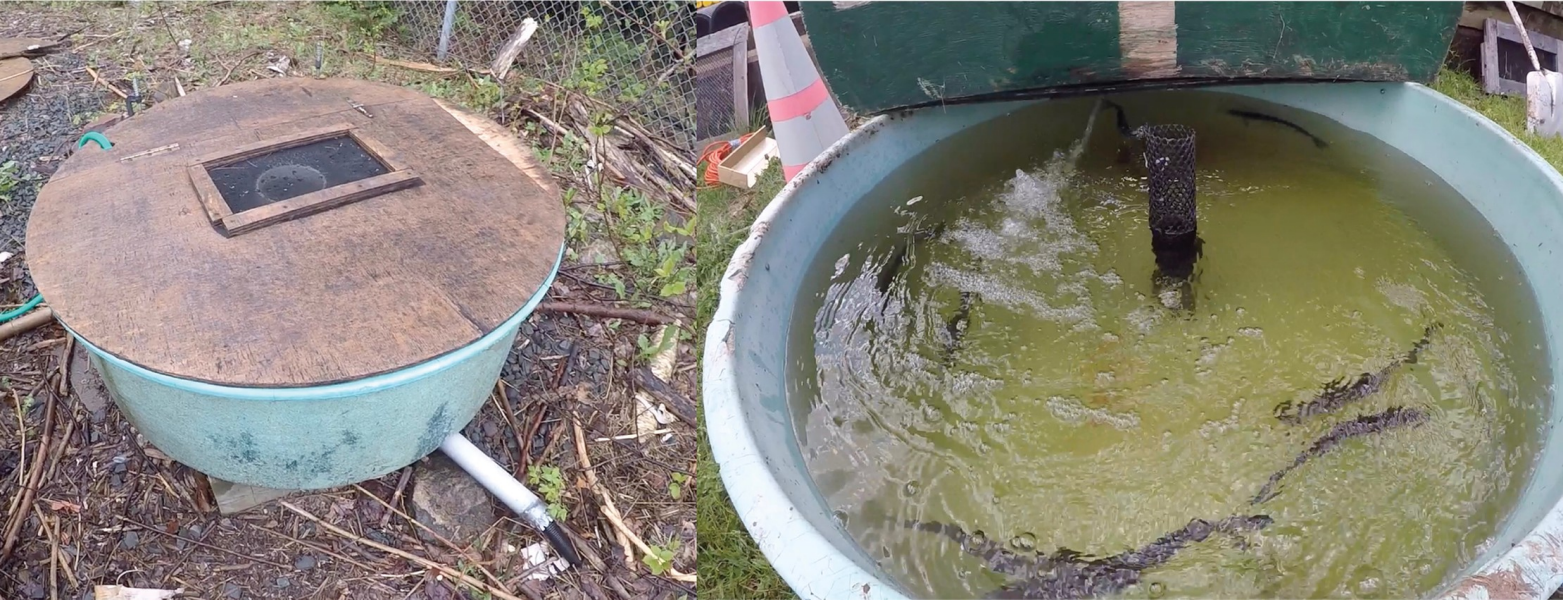
Pictures of holding tank (270 L) used to retain alewives during field trials at the White Rock Fish Ladder, Gaspereau River, Nova Scotia. Both the holding and recovery tanks were analogous and located outdoors near the fish ladder on Nova Scotia Power Inc property.

Alewives from the Gaspereau River were captured at the White Rock Fish Ladder, located approximately 7.5 km above the head of the tide (Fig 1). Fish were captured through dip netting and transferred to an outdoor, circular holding tank (270 L capacity; Fig 2). Water was continuously cycled through the tank by a pump submerged in the fish ladder, thereby maintaining a steady supply of oxygenated water at a temperature equivalent to the river environment, as well as creating a circular current to facilitate the alewives’ swimming behaviour. An internal standpipe at the center of the tank controlled the water level, with the outlet flow directed back into the river. Temperature in the river and holding tank was measured at the start of each tagging day using a hand-held thermometer. Temperature of the anaesthetic baths was maintained within 2˚C of the river temperature. All fish handling was done in accordance with animal care guidelines to minimize descaling [33]. Surgical instruments were soaked in a 10% betadine sterilizing solution and rinsed with sterile water before and after each use.

### 2018 anesthetic trials

For the anesthetic trials, 30 alewives were anesthetized with tricaine methanesulfonate (MS-222) between May 23 and 25: 10 fish were subjected to an anesthetic immersion bath at a concentration of 60 mg/L, 10 to a concentration of 100 mg/L, and 10 to a concentration of 200 mg/L. All anesthetic baths were buffered with sodium bicarbonate (NaHCO_3_) at a 1:2 ratio. An additional 10 fish were used as untreated controls to compare against the behaviour of treated fish after recovery. Animals were held until they reached surgical anaesthesia, characterized by a complete loss of equilibrium and reactivity to stimuli [34]. Anesthesia induction time, and the time to regain vertical equilibrium (recovery time) were used as the response variables. All animals were released upon completing the trials. Morphometric measurements of sampled fish are outlined in Table 1.

**Table 1:** Average weight, length and water temperature parameters of alewife trial groups across the 2018 and 2019 field seasons.

Treatment	N	Fork length (mm) Mean ± SD	Weight (g) Mean ± SD	Temperature (˚C) Mean ± SD
2018 pre-spawners, 60 mg/L anesthesia only	10	249 ± 10	196 ± 24	14 ± 0.0
2018 pre-spawners, 100 mg/L anesthesia only	10	247 ± 12	205 ± 35	15 ± 0.5
2018 pre-spawners, 200 mg/L anesthesia only	10	234 ± 13	146 ± 33	15 ± 0.0
2018 pre-spawners, incision-only	8	248 ± 7	188 ± 22	16 ± 0.6
2018 pre-spawners, Tagging without suturing	11	232 ± 12	173 ± 21	15 ± 0.5
2018 pre-spawners, Tagging with suturing	10	249 ± 12	183 ± 47	16 ± 0.5
2019 pre-spawners, tagging with suturing	52	251 ± 10	218 ± 29	10 ± 0.9
2019 post-spawners, tagging with suturing	44	248 ± 12	170 ± 27	17 ± 1.3

### 2018 incision closure trials

A concentration of 200 mg/L was chosen for the following trials (see results for details); 29 alewives were captured from the ladder and assigned to one of three trial groups: tagging with suture closure (n = 10), tagging without closure (n = 11), and incision-only (no tag or closure; n = 8) (Table 1). The incision-only group was used to try to separate the effects of tag burden from the surgical procedure itself. Ten additional alewives were used as untreated controls. Tagging took place between 25 May and 7 June; water temperature during this time ranged from 15–16˚C. In addition, 5 post-spawned fish were captured and tagged on June 13, but no more fish could be captured due to the needs of Nova Scotia Power Inc. for managing water levels at their generation station. These fish were removed from the statistical analysis to avoid spawning condition as a confounding variable, but qualitative observations from their tagging will be discussed.

**Fig 3:**
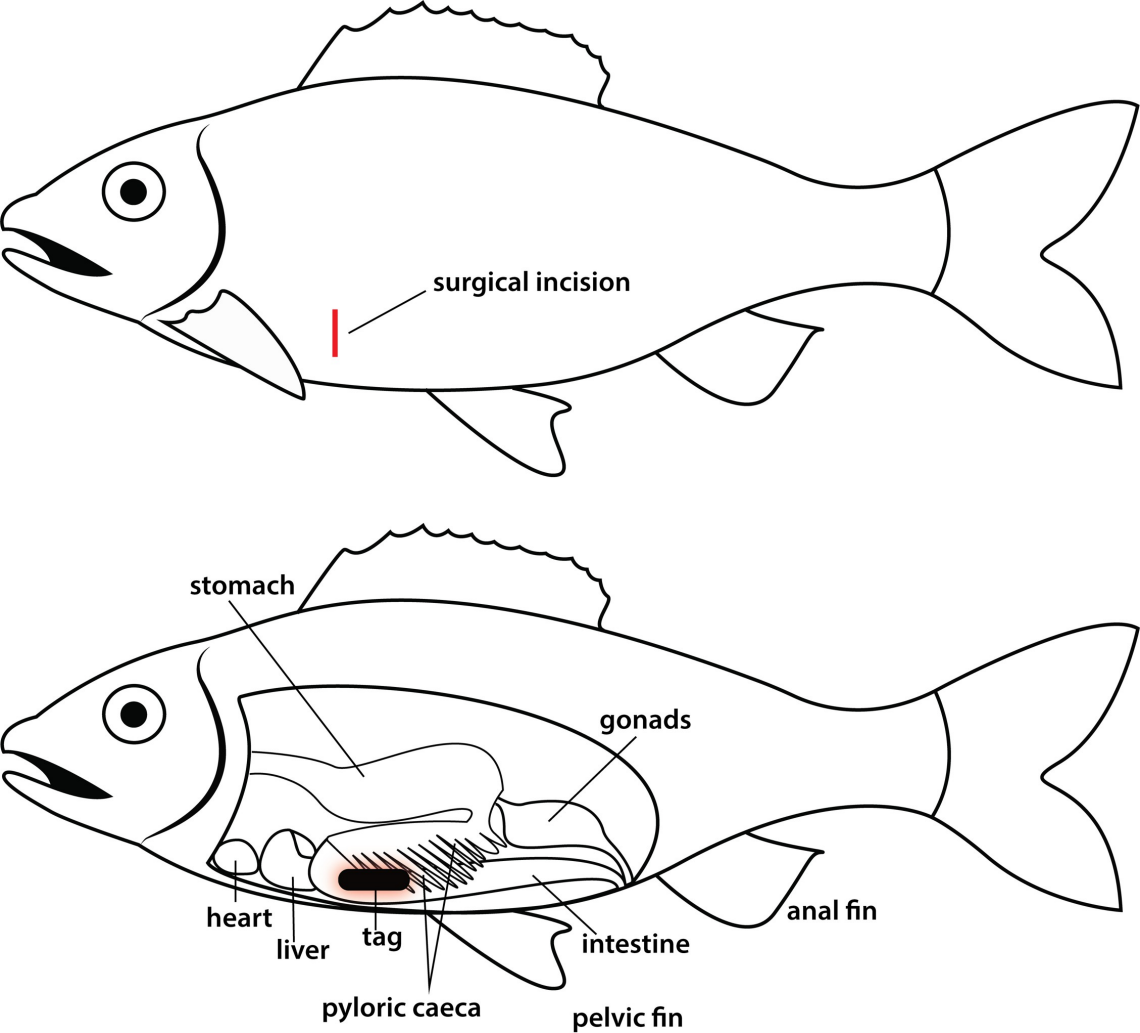
**Position of the incision in red (top) and tag (bottom) following surgical implantation on the right lateral side of the fish, with internal anatomy shown**.

The tagging protocol was adapted from methods described in previous studies on alewife [18,19], Pacific herring [16,17], and Atlantic salmon (*Salmo salar*) smolts [35], and the location of the incision site was based on passive acoustic transponders (PIT) tagging practices [36]. Fish were anesthetized with a buffered solution of 200 mg/L MS-222, and morphometric measurements (sex, weight and fork length) were recorded (Table 1). Fish were then placed in a surgery cradle supplied with an aerated anaesthetic solution at 100 mg/L MS-222 to maintain adequate sedation, and tagged with dummy Vemco/Innovasea V5, high residency (HR) acoustic transmitters. These tags measure 12.7 mm in length by 5.8 mm in diameter and weigh 0.77 g (0.46 g in water), resulting in a tag burden of 0.3% to 0.6%. An incision just large enough to allow passage of the tag was made between the ribs on the right side of the fish, at the caudal extent of the pectoral fin and 2–3 rows of scales dorsal to the ventral midline. This location was selected because the body wall there is thin, and the wound is less likely to affect swimming ability [2]. The position also granted easier access and more control for the tagger than a typical ventral midline incision, given the alewife’s compressed body form. Fish from the incision-only group were recovered; for the remaining fish, a dummy V5 tag was gently inserted and pushed forward into the peritoneal cavity in a cranial direction, so as to be positioned above the pyloric caeca (Fig 3).

Eleven of the tagged animals were then allowed to recover without suturing the incision site. For the remaining 10 fish, incisions were closed with two simple interrupted sutures using 4/0 Ethicon monofilament (reverse cutting, 1.5 metric, 45 cm, PS-2 18 mm, 3/8 circle needle). We recorded the handling time and the time it took each animal to recover vertical equilibrium upon release into the recovery tank. Due to a lack of indoor facilities and equipment required for the long-term holding of animals [37] we were limited in retaining post-surgery fish for a maximum period of 72 h. Animals were visually assessed ~20 min after tagging, and once daily for the next 72 h for signs of acute stress, such as struggling to maintain vertical equilibrium for an extended period after tagging, or showing extensive signs of infection (>20% of body surface). At the end of the experiment, tagged alewives were euthanized through anesthesia overdose and necropsied to recover tags and assess internal effects of surgeries. Instances of hemorrhage or bleeding (either externally or internally), damage of internal organs, or shifts in the tag position were noted, and the information was used to inform future tagging trials, however no overall impairment score was assigned, and these data were not further analyzed. All remaining fish (incision-only and control) were released back into the White Rock Fish Ladder.

### 2019 RAMP assessment

In 2019, the tagging procedure was replicated to examine behavioural effects of tagging in more detail. A total of 96 adults (52 ripe, 44 spent) were captured between 19 May and 25 June 2019 from the White Rock Fish Ladder. Water temperature ranged from 9 to 11˚C in May (start of the run) to 15 to18˚C in June (end of the run). Tag burden ranged from 0.3% to 0.7%. Each fish was sexed, weighed and measured after being anesthetized with 200 mg/L of MS-222 (Table 1), and tagged as per the pilot trials. All incisions were closed with two simple, interrupted sutures.

Following surgeries, fish were recovered for 5 min in a closed, aerated 100 L tank to allow recovery from anesthesia. When the lid of the tank was removed, the behavioural condition of the fish was assessed based on RAMP methods previously described for other taxa [e.g., 29]. The reflexes tested included: ventilation, orientation, swimming vigour, light response and tactile response. Each reflex was assigned a score of 0 if non-impaired, and 1 if impaired. Ventilation was considered unimpaired if the fish exhibited regular opercular movement for 30 s. An unimpaired orientation response was noted if the fish maintained vertical equilibrium in the water, and unimpaired swimming consisted of sustained, regular movement for at least 30 s. A startle response was expected for both the light (lifting tank lid) and tactile (tail grab) stimuli, in which unimpaired fish show rapid forward motion in response to the stimuli. Total RAMP scores for individuals were calculated as a proportion of the five measured reflex scores (0 = no reflexes impaired; 1 = all reflexes impaired). Fish were visually inspected for signs of acute stress or mortality ~20 min following the final surgery, and after 24 h, however longer holding was not possible.

### 2019 Necropsy assessment

After being held for 24 h, alewives were euthanized by anesthetic overdose, and a necropsy assessment was conducted. No mortalities or instances of tag shedding were observed during this holding period. Fish were assessed based on the condition of the incision site, internal organs, and tag position within the body cavity (Table 2). Each condition was assigned a score of 0 or 1, and a total necropsy index was calculated as the proportion of physical parameters that had been impaired or damaged.

**Table 2:** Physical index examined at necropsy.

Category	Unimpaired (0)	Impaired (1)
External	No scale loss or lesions	Scale loss >20%, lesions, hemorrhage or bleeding around mouth, eyes or fins
Incision site	Normal, with no redness or swelling	Evidence of infection, hemorrhage or severe bleeding externally or internally
Internal organs	Normal, with no redness or swelling	Focal or general discolouration
		Swelling
		Hemorrhage / bleeding
		Puncture or other damage
Tag position	Above the pyloric caeca	Tag shifted from initial position above the pyloric caeca forward or backward into the body cavity

Each category was assigned a score of 0 if not impaired, and 1 if any impairment was observed. Total index score calculated as the proportion of impaired categories (0 if all normal, 1 if all impaired).

### Data analysis

Recovery times between anesthesia and between tagging treatment groups were compared using one-way Analysis of Variance (ANOVA) with Tukey's Honestly Significant Difference (HSD) post hoc test at an alpha level of .05. Recovery times for both were shown to be normally distributed based on the Shapiro–Wilk test for normality (P > 0.05). RAMP and necropsy scores were modelled individually using a Generalized Linear Model with a quasibinomial error distribution. Fish weight, sex, water temperature and handling time were used as predictor variables for the RAMP model, while weight, sex, and spawning condition were used as predictor variables for the necropsy model. Collinearity was assessed using Spearmen’s rank correlation tests, and only variables with a correlation not exceeding 0.5 were selected. Model fit was assessed by examining graphical plots of residuals versus fitted values for temporal and spatial dependency or serial autocorrelation, as per Zuur et al. (2009), as AIC and BIC are not defined for quasibinomial models. All analyses were undertaken in R (version 3.6.1).

## Results

### 2018

**Fig 4:**
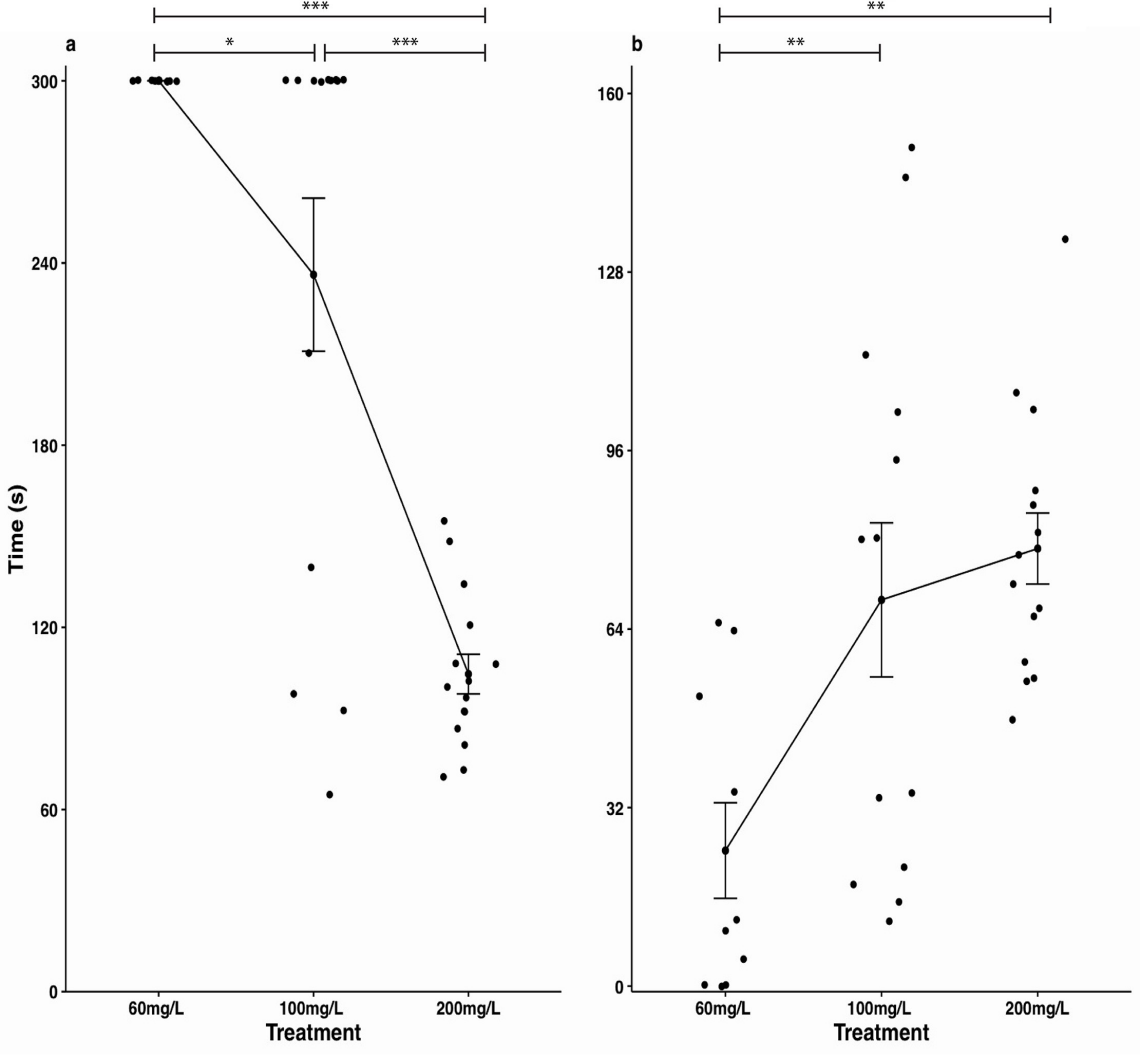
**Anesthesia induction (a) and recovery (b) times at 15˚C for MS-222 concentrations of 60 mg/L, 100 mg/L and 200 mg/L.** Times were capped at 5-minutes. * P < 0.05; ** P < 0.01; *** P < 0.001 one-way ANOVA with Tukey's Honestly Significant Difference (HSD) post hoc test.

The optimal concentration of MS-222 at 15˚C (average temperature during the tagging period) was determined to be 200 mg/L. At this concentration, both anesthesia induction and recovery times were consistently below 5 min (Fig 4). Induction time was significantly lower than both 60 mg/L and 100 mg/L (F_2,34_ = 33.41, P < 0.001); recovery time was longer than 60 mg/L (F_2,34_ = 7.228, P = 0.002) and did not differ from 100 mg/L (F_2,34_ = 7.228, P = 0.785), but there was less spread in the measurements. All animals from the 60 mg/L treatment group took longer than 5 min to be anesthetized, and several did not reach a full state of unconsciousness, leading to immediate recovery following handling and returning to water.

**Fig 5:**
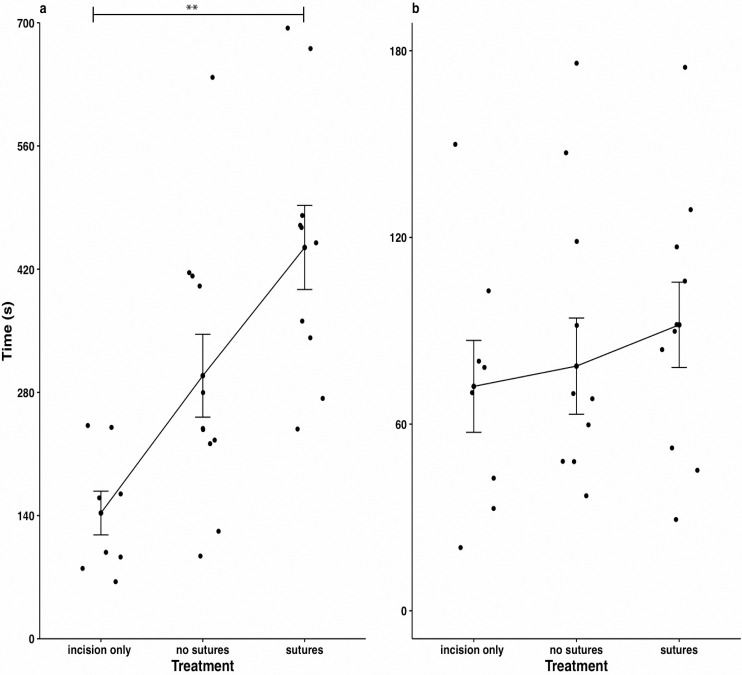
**Treatment (a) and recovery (b) times of incision-only, sutured and non-sutured trial groups tagged with dummy V5 acoustic tags at an average temperature of 15˚C.** ** P < 0.01 one-way ANOVA with Tukey's Honestly Significant Difference (HSD) post hoc test.

Despite a longer time required for suturing compared to non-suturing and incision-only treatments (Fig 5), recovery time for the suture treatment group did not significantly differ from the no closure (F_2,25_ = 0.406, P = 0.801), and incision-only (F_2,25_ = 0.406, P = 0.662) groups. No instances of tag loss were observed after 72 h post-surgery in any of the treatments. Most animals recovered vertical equilibrium within 2 min after being placed back into flowing water, with an average recovery time of 68 s (40 s SD). Swimming behaviour was generally indistinguishable from control fish within 5 min of tagging. Incision edges remained open in all non-sutured fish, with 2 fish showing signs of redness or bleeding at the end of the holding period. Early signs of fungal infection (presumably *Saprolegnia* sp.) were noted in the post-spawned fish. One alewife from this group was found dead at the end of the holding period; the fish was necropsied to assess the cause of mortality.

### 2019

**Fig 6:**
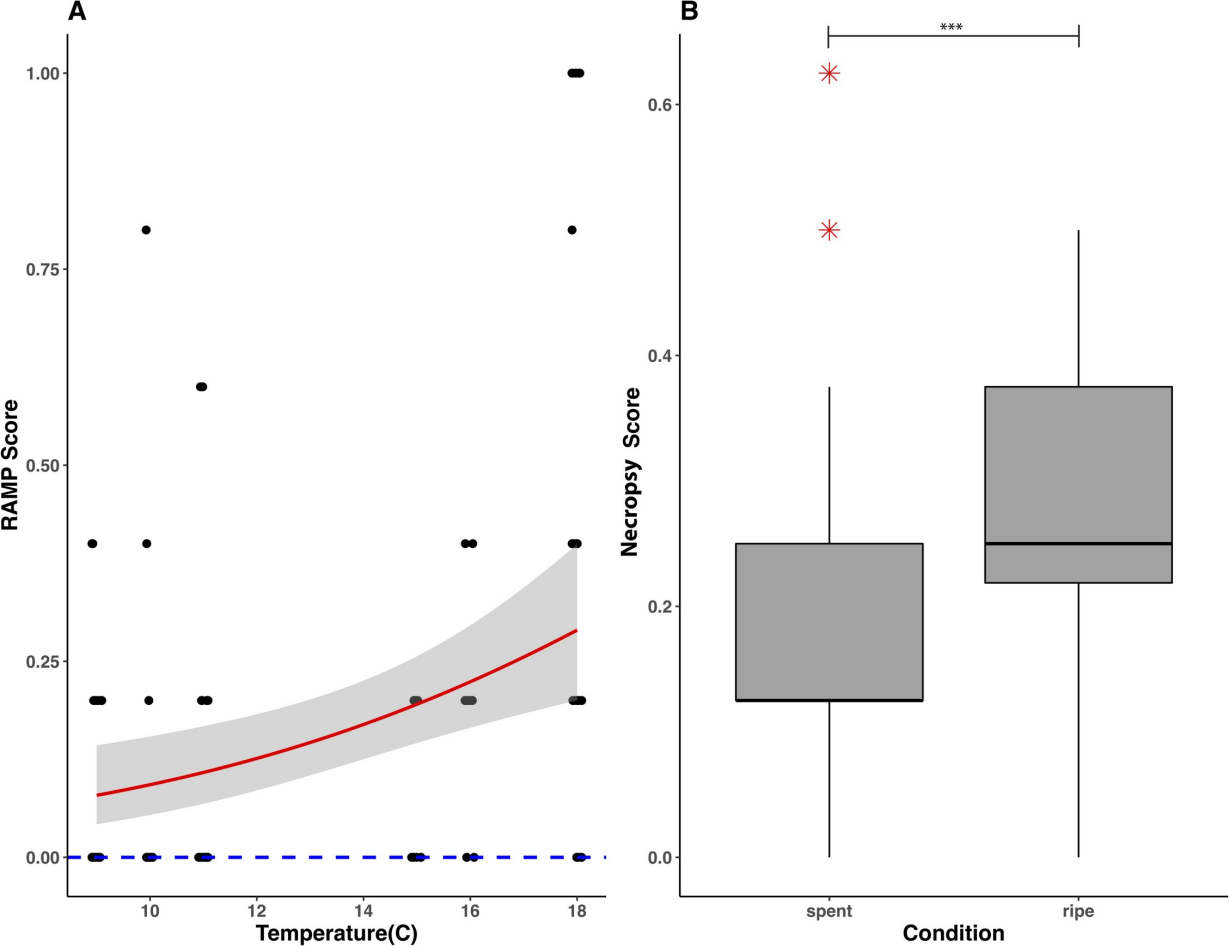
**(A) Change in RAMP scores as a function of water temperature during tagging. Red line represents general linear regression with quasibinomial error distribution and 95% confidence intervals. Zero-intercept shown by the dashed blue line. (B) Boxplots showing median and quartile range of physical impairment scores for pre and post-spawned alewives, with whiskers at 1.5 IQR of the upper/lower quartiles.** Outliers are shown in red. *** P < 0.001 GLM.

Average RAMP scores 5 min post-surgery were 0.2 ± 0.3 out of 1, with 4% (n = 4/96) of fish experiencing an impairment of all reflexes. Response to light stimulus was the most commonly affected reflex. Significant differences were not detected for sex (t-value = -0.313, P = 0.76), size (t-value = 0.505, P = 0.62), spawning condition (t-value = 1.836, P = 0.07) or handling time (t-value = -1.775, P = 0.08), but a higher proportion of reflexes was impaired at temperatures above 12˚C (t-value = 2.656, P = 0.01) (Fig 6). Average necropsy scores recorded 24 h post-surgery were 0.2 ± 0.1 out of 1, and no animal received a full physical impairment score in the necropsies. The most common internal injury observed was a puncture of the gonads by the tag. This was only observed in pre-spawned (ripe) fish, and spawning condition was the only significant predictor of necropsy scores (t-value = 3.962m, P < 0.001) (Fig 6).

## Discussion

This study demonstrated that the acoustic tagging of alewife is feasible. We observed high recovery success in pre and post-spawned animals, and at lower temperatures. The use of sutures did not have a significant effect on recovery time. However, because long-term monitoring was not logistically possible we did not test for latent effects. The protocol outline above remains to be fully validated through long-term holding or field trials.

A concentration of 200 mg/L of MS-222 achieved the best balance between anesthesia induction and recovery times in our trials. At 60mg/L induction was prolonged, and some fish were not reliably anesthetized, such that they began twitching while being handled, and subsequently recovered very soon after being placed in the recovery tank. At 100 mg/L there was a lot of individual variation in both induction and recovery times, with some fish seemingly more affected by the dose than others. The concentration of 200 mg/L consistently produced reliable results, and was therefore chosen for all subsequent trials.

One often debated aspect of animal tagging is the use of sutures to close an incision. On one hand, the use of sutures is generally recommended when performing surgeries as it helps to reduce tag shedding and infection [38,39]. However, sutures have also been shown to cause adverse effects like tissue inflammation [38,40]. Some researchers have started to move away from suturing when tagging fishes, which is enabled by smaller tag sizes. For example, PIT tags, which are generally smaller than acoustic tags, are injected into the body cavity through a non-sutured incision [36]. This method improves survival and reduces effects of suturing, such as inflammation or fungal infection at the tagging site in some species [41–43], although in other cases tag retention is reduced [44]. Small acoustic transmitters can similarly be injected intracoelomically [36], and though adverse effects have been observed [45], overall, the downsizing of acoustic transmitters is becoming the preferred choice, particularly for smaller fishes [46,47].

This study used V5 acoustic tags. These transmitters are substantially smaller than models used in previous studies on clupeids [16–19] and reduce the tag burden on animals, as well as potentially eliminate the need for suturing. Our pilot trials tested the feasibility of not suturing incisions in alewife. No statistical differences in recovery times were noted between sutured and non-sutured fish, but most wounds that were closed with sutures showed some approximation of the wound edges after 3 days of holding, while non-sutured wounds remained open. Instances of fungal infections were seen in 5 individuals in 2018, all of which had non-sutured incisions, suggesting that suturing might be better for reducing post-tagging infection rates in freshwater. However, in 2019, ~50% of animals showed signs of bleeding or of inflammation around the sutures during necropsies conducted 24 h after tagging. While some inflammation is expected after such a short time interval, this prevents us from drawing any conclusions about the use of sutures in this species. Because our study was limited to a short time, we could not assess the effects of suturing on wound healing, feeding, growth, and long-term survival in our species. The fish that survived in this experiment could still suffer delayed mortality or tag expulsion [7,16]. Therefore, we did not have sufficient information to confidently show that there would be no drawbacks from not suturing alewife. For this reason, sutures were still used in the 2019 trials. Future studies would help to validate our method through long-term holding, and could test a surgical protocol that does not use sutures for this species.

We did not find any allometric differences in RAMP scores in post-surgery alewives, contrary to observations in other taxa [e.g., 48]. It is likely that the alewives captured in this study did not have a sufficient size range to observe these differences, as fork length only ranged from 23 to 28 cm. The only significant predictor of reflex impairment was water temperature. A cumulative 3% (n = 3) mortality was observed in the 2019 alewife tagging trials, with all mortalities occurring on the last tagging day (June 25), when water temperatures were at their highest (18˚C). In this group, several fish took longer than expected to recover vertical equilibrium (> 5 min). However, each trial group was visually inspected ~ 20 min after the last surgery, and no instances of mortality or fish struggling to maintain vertical equilibrium were observed at this time, suggesting latent effects of thermal stress that were not manifested in behavioural changes. Numerous studies have reported a correlation between water temperature and tagging success, with fish suffering increased physiological stress, decreased immune response, and higher mortality at higher temperatures [49–51]. Water temperature also affects anesthesia induction and recovery times, so the time it took fish to reach surgical anesthesia varied between days, but was generally still below 5 min [34]. Post-mortem assessments did not reveal any significant internal injuries in the deceased fish compared to those that had been euthanized, suggestive that tagging error was not the cause of these deaths. However, some animals still showed zero reflex impairment even at 18˚C. It is possible that physiological characteristics make certain animals more susceptible to the effects of temperature. This was not observed in our study, but further research is required to examine temperature-induced stress in clupeids.

Because reflexes are linked to underlying physiological pathways [23], reflex impairment is used to predict the fate of individuals. In Coho salmon (*Oncorhynchus kisutch*), individuals with greater reflex impairment experienced higher rates of migration failure [24], and a significant relationship between delayed mortality and RAMP scores was observed in yellowtail snappers (*Ocyurus chrysurus*) [52]. A trend toward reduced rate of movement was also observed in white sturgeon (*Acipenser transmontanus*) that had poor outcomes in RAMP test [28]. A similar relationship can be expected for alewife, with individuals scoring lower on the impairment scale having a higher chance of survival. In species where the correlation between reflex impairment and mortality was measured, the relationship is generally sigmoidal, with RAMP initially increasing without concomitant mortality, but further increase being associated with increasing mortality [23,53]. No RAMP curves have been made for clupeids, so it is difficult to estimate what proportion of fish tagged in this study might be expected to experience delayed mortality. However, research in other species can offer some speculative suggestions. Of the studies we were able to find, the proportion of impaired reflexes at which a 50% mortality was observed ranged from ~ 0.1 in Coho salmon smolts [25] to ~0.7 in rock sole (*Lepidopsetta bilineata*) and halibut (*Hippoglossus stenolepis*) [23,52,53]. Assuming that alewife fall within this range, we might expect between 6% and 23% of our fish to experience latent mortality. Future research using long-term holding or telemetry tracking to estimate mortality would be valuable for developing a species-specific RAMP curve, which could then be applied to predicting survival of alewife in telemetry studies.

Following the necropsies, higher scores were observed in pre-spawned fish, which generally appeared more robust and quicker to recover from tagging compared to post-spawners but had a higher chance of gonad damage from tagging. It is not clear whether the puncture of gonads by a tag could have long-lasting impacts on fish survival. Based on evidence from PIT tagging studies on ripe alewife, this injury is not likely to be life-threatening, as many fish continue to be detected several years after tagging [42]. Necropsies performed after long-term laboratory studies in other taxa reveal that transmitters generally become encapsulated in soft tissue, and often adhere to the visceral organs or body wall without affecting animal growth or survival [16,22,50]. However, the effects on reproduction are poorly understood. Tagging trials in wild rainbow trout (*Oncorhynchus mykiss*) revealed no difference in gonad development between tagged and control fish [54], but in another study, while onset of sexual activity did not differ, captive-bred steelhead females retained more eggs during spawning and had higher mortality compared to nontagged females [55]. We recommend future laboratory studies to assess latent changes in growth rate and behaviour from sublethal tagging damage in clupeids. To mitigate this during field studies, we recommend inserting the tag horizontally at a 30–45˚ angle in order to ensure that it is pushed forward and not down into the body cavity.

Five animals in the 2018 trials were infected with a freshwater fungus, of which one was found dead on the third day of holding, and the other four were euthanized for further examination. All five fish were post-spawners, tagged on the last trial day. Necropsies did not reveal any internal damage, other than the growth of greyish-white patches of filamentous mycelium on the alewives’ bodies, which were assumed to be a species of *Saprolegnia*. *Saprolegnia* is a zoosporic oomycete commonly found in freshwater environments worldwide, and a leading cause of disease in fish hatcheries [56]. There was no difference in housing conditions, holding time, or fish size between this and other treatment groups in 2018, and water temperature was only one degree warmer than the previous tagging day, leading us to believe that a weaker immune system as a result of spawning, and/or the longer time spent in freshwater were the primary contributing factors to why *Saprolegnia* was only observed in this group. Post-spawned fish may be particularly susceptible to infection due to decreased immunity caused by the stress of spawning [57], and perhaps their condition was exacerbated by water temperature. However, many of the pre-spawners were tagged at 16˚C and did not experience *Saprolegnia* infection. The upper lethal temperature limit previously reported for this species is > 23.5˚C [58], possibly up to 31–34˚C [59], which is higher than the 17˚C recorded on the tagging day. These fish would also have spent more time in freshwater, which could increase their chances of becoming infected, as *Saprolegnia* is a freshwater mold that is not tolerant of high salinities, and would therefore not be present in the marine or estuarine environment [60,61]. No infections were seen in post-spawners in the 2019 trials, although this could be a result of shorter holding times. Overall, in post-spawned fish the fungus should clear once the animals return to sea. As no other instances of infection were observed in 2018 or 2019, it is not expected to be a significant stressor during tagging studies in this area.

We noted that clupeids have certain physiological requirements that must be considered during the tagging process. First, alewives appeared to be better adapted for ram ventilation than buccal pumping, and therefore required a continuous, strong flow of water to maintain regular swimming and ventilation during recovery. Fish that were submersed in stagnant water after an anesthetic treatment took longer to recover vertical equilibrium, even if they had not been subjected to other manipulations. Second, alewives descale very easily, therefore care should be taken to minimize contact with hard surfaces or abrasive materials. Other factors like surgeon experience, environmental parameters (e.g. water temperature, pH, dissolved oxygen), tag size, suture material, and physical characteristics of the animals can all influence the outcomes of tagging [51]. It is paramount that researchers continue to conduct tag evaluation studies, especially when working with new tag models, or poorly-studied species. As tagging technology continues to improve toward minimizing transmitter size, new surgical methods can emerge that will further decrease tagging effect.

## Conclusions

The tagging method developed for this study, which uses a combination of small tags, a lateral tag insertion, and flow-through anesthetic during surgeries, was effective in alewife examined. Despite their sensitivity to handling, fish responded well to the procedure, with most recovering full functionality of their reflexes within 5 min following tagging, and 97% of all tagged individuals surviving into the following day. Post-mortem internal examinations revealed few instances of physical damage that would be considered life-threatening. Continued research is needed to assess whether tagging can cause long-term physical and behavioural effects, and whether there is a thermal threshold above which deleterious effects consistently occur, to ensure that the tracking data are biologically relevant. Given the overall success of the tagging trials, we recommend the use of this protocol in future acoustic tracking studies of clupeids.
